# A Fast-Track Referral System for Skin Lesions Suspicious of Melanoma: Population-Based Cross-Sectional Study from a Plastic Surgery Center

**DOI:** 10.1155/2016/2908917

**Published:** 2016-07-20

**Authors:** Reem Dina Jarjis, Lone Bak Hansen, Steen Henrik Matzen

**Affiliations:** Department of Plastic Surgery & Breast Surgery, Zealand University Hospital, Sygehusvej 10, 4000 Roskilde, Denmark

## Abstract

*Introduction*. To minimize delay between presentation, diagnosis, and treatment of cutaneous melanoma (CM), a national fast-track referral system (FTRS) was implemented in Denmark. The aim of this study was to analyze the referral patterns to our department of skin lesions suspicious of melanoma in the FTRS.* Methods*. Patients referred to the Department of Plastic Surgery and Breast Surgery in Zealand University Hospital were registered prospectively over a 1-year period in 2014. A cross-sectional study was performed analyzing referral patterns, including patient and tumor characteristics.* Results*. A total of 556 patients were registered as referred to the center in the FTRS for skin lesions suspicious of melanoma. Among these, a total of 312 patients (56.1%) were diagnosed with CM. Additionally, 41 (7.4%) of the referred patients were diagnosed with in situ melanoma.* Conclusion*. In total, 353 (63.5%) patients had a malignant or premalignant melanocytic skin lesion. When only considering patients who where referred without a biopsy, the diagnostic accuracy for GPs and dermatologists was 29% and 45%, respectively. We suggest that efforts of adequate training for the referring physicians in diagnosing melanocytic skin lesions will increase diagnostic accuracy, leading to larger capacity in secondary care for the required treatment of malignant skin lesions.

## 1. Introduction

Cutaneous melanoma (CM) is an aggressive tumor of the skin melanocytes. It is a growing health concern with a globally increasing incidence in fair-skinned populations over the last few decades [1]. The cause of CM is probably multifactorial but for the most part attributed to an increase of exposure to UV radiation [1, 2].

To minimize delay between presentation, diagnosis, and treatment of CM, the Danish Health and Medicines Authority implemented a national fast-track referral system (FTRS), which also includes that the surgical management of primary melanoma is according to national clinical guidelines. Other skin malignancies, such as basal cell carcinoma (BCC) and squamous cell carcinoma (SCC), are not intended to be referred through this system. The aim of the national FTRS was “*that cancer patients should experience a well-organized, comprehensive process without undue delay in connection with the clinical evaluation, initial treatment and rehabilitation and palliative care, with the aim to improve the prognosis and quality of life for patients*” [3].

The Department of Plastic Surgery & Breast Surgery, Zealand University Hospital, provides a regional health service to a population base of approximately 820.000 inhabitants in Zealand Region in Denmark, representing nearly 15% of the country's total population. The incidence of melanoma in Zealand Region in a five-year period in 2010 to 2014 increased from 245 to 445 patients, including MIS [4]. In the recent years, this incidence has amounted to approximately 14-15% of the diagnosed CM in the total population, and this regional incidence is slightly increasing correspondingly with the national incidence in Denmark [5].

In the Danish health care system, the general practitioners (GPs) function as gatekeepers for consultant dermatologists, plastic surgeons, and plastic surgery departments, where treatment of CM is performed. The Danish national FTRS for skin lesions suspicious of melanoma implies that a patient who presents with a lesion suspicious of CM is offered an excision biopsy in secondary care within six days. This allows confirmation of the diagnosis within two weeks and permits the second stage of definitive wider excision and sentinel lymph node biopsy (SLNB) to be based on tumor characteristics such as the Breslow thickness, mitotic activity, or presence of ulceration, which is then offered within nine days. If SLNB is positive for metastasis, the patient is offered the third stage comprising radical lymph node dissection within two weeks.

Our plastic surgery department receives referrals regarding lesions suspicious of melanoma from GPs, dermatologists, private plastic surgery clinics, or other specialties for clinical evaluation and treatment. If a biopsy is made prior to the referral, the patients are offered further treatment in the department depending on the histological examination of the biopsy.

The aim of this study was to analyze the referral patterns of suspicious melanocytic skin lesions to our department in the FTRS. We hypothesize that a relatively large proportion of the referrals will present with skin lesions other than CM, leaving room for improving diagnostic accuracy in the primary health care sector. Therefore, the objective was to characterize the referrals in the FTRS of suspected or classified CM, in order to clarify from which health services the referrals originate and how many of the referred patients had in situ melanoma (MIS), invasive cutaneous melanoma (CM), non-melanoma skin cancer (NMSC), or benign lesions.

## 2. Methods

### 2.1. Study Design and Setting

Patients referred in the FTRS to the Department of Plastic Surgery and Breast Surgery in Zealand University Hospital, because of a lesion suspicious of CM or with a biopsy-verified CM, were registered prospectively in a population-based cross-sectional study of the Zealand Region consisting of approximately 820.000 inhabitants in Denmark.

### 2.2. Participants

This study included patients over a 1-year period in 2014, who had undergone surgical biopsy in general practice, by dermatologists, in private plastic surgery clinics or in other medical specialties and who were referred to our department for further treatment. Patients who were referred by the above health services because of a suspicious lesion without a biopsy prior to the referral were also included. In the first visit to the outpatient clinic, the patient was examined clinically and by manual dermatoscopy, and a total body skin examination was performed. Also, the patient was offered an immediate excision biopsy under local anesthesia, if a biopsy was not performed prior to the referral. The patients were scheduled after two weeks for suture removal and results of histology. Patients who did not show up to the consultation or who did not want to have an excision biopsy performed and patients with metastatic melanoma of unknown primary origin were excluded from the study.

### 2.3. Variables

Variables are patient age, sex, anatomical location of the lesion, tumor characteristics, and from which health services the referrals originated were registered, along with whether or not a biopsy had been performed prior to referral. All cases were coded as in situ melanoma (MIS), invasive cutaneous melanoma (CM), non-melanoma skin cancer (NMSC), or benign lesions.

The histological examinations of all the tumor biopsies were conducted at the Department of Pathology, Roskilde University Hospital.

### 2.4. Statistical Methods

All data were tested for distribution of normality and treated statistically accordingly. Comparison between groups was tested by two-tailed* t*-test and in the case of skewness or few numbers among groups the Mann-Whitney *U* test for nonparametric data was performed. Statistical significance was set at *P* < 0.05. All data were analyzed using the statistical program PAST (ver. 3.09).

## 3. Results

### 3.1. Participants

A total of 565 patients were prospectively registered as referred to the center in the FTRS for skin lesions suspicious of melanoma (Table 1). Of the referred patients, two patients never showed up, one patient did not eventually want a biopsy taken, and seven patients had metastatic melanoma of unknown primary origin demonstrated by PET-CT scan. These nine patients were excluded from the database.

### 3.2. Data

Most of the patients (393; 70.7%) were referred due to a suspicion of CM and had an excision biopsy taken on the first visit, whereas 159 (28.6%) patients were referred due to a biopsy-verified CM.

A total of 312 patients (56.1%) were eventually diagnosed with CM, significantly more females than males (*P* < 0.05). The rest of the referred patients were diagnosed with MIS (41; 7.4%), NMSC (29; 5.2%), or various benign skin lesions (171; 30.8 %) with no significant difference between males or females (Table 1). Most referrals (441; 79.3%) were from dermatologists and only five from plastic surgeons (Table 2). Of the 393 patients without a biopsy prior to the referral, 55 patients were referred by GPs, 321 patients by dermatologists, and 17 patients by other specialties. Of the patients referred by GPs and dermatologists, a total of 16 and 145 patients, respectively, were diagnosed with CM (Table 2), leading to a diagnostic accuracy of 29% for GPs and 45% for dermatologists.

When Breslow thickness was analyzed according to age groups and sex (Table 3), the largest proportion of both sexes was in the group of thin melanomas (≤1 mm). However, this number was only statistically significant for females (*P* = 0.044 versus males *P* = 0.47). There was a tendency for a relatively larger distribution of males in the middle group of CM thickness, compared to females, but this finding was not statistically significant (Table 3). There were no major differences in distribution on Breslow thickness of the referrals originating from GPs or dermatologists when biopsy is performed prior to and after referral (Table 4). However, there might be a tendency towards a larger proportion of thin melanomas excised by GPs.

All of the included patients were seen after referral within the recommended time period and there was no difference in timing according to who was referring the patient. Furthermore, all patients diagnosed with CM had a definitive wider excision performed based on tumor characteristics in accordance with national guidelines for treatment of melanoma, including SLNB and radical lymph node dissection, if these were indicated.

## 4. Discussion

This is the first population-based cross-sectional study in Denmark that provides insight into the diagnostic accuracy of referrals of patients with skin lesions suspicious of melanoma, and the referring physicians' ability to differentiate between CM, NMSC, and benign lesions. Of all the referred patients, a total of 312 patients (56.1%) were diagnosed with CM and 41 patients were diagnosed with MIS (7.4%), whereas 200 patients had NMSC or benign lesions (35.9%). The FTRS can improve rapid access for patients with CM, but only when used appropriately due to increased education, clear communication, and improved technology for consistent detection of cancers [6]. A study has demonstrated that physicians can benefit from using dermatoscopy to differentiate between pigmented lesions, and this could possibly reduce the unnecessary referrals [7]. However, this requires proper training and regular use of dermatoscopy in order to improve from its supplementary assessment and therefore it is questionable whether GPs have the time required to gain sufficient experience in using dermatoscopy.

In our presented data, most referrals (441; 79.3%) were from dermatologists and only five from plastic surgeons (Table 2). This fact reflects that in the Zealand Region there are several practitioners in dermatology and none in plastic surgery. Also, this might indicate that most of the patients were referred to a dermatologist by the GPs initially, and these were further referred to our department in the FTRS because of continued clinical suspicion of CM (321; 57.7%) or because a biopsy performed by the dermatologist had verified the diagnosis of CM (106; 19.1%). When evaluating the diagnostic accuracy, the referring GPs had a lower accuracy compared to dermatologists (29% versus 45%), which is not unexpected since most GPs in general do not use dermatoscopy and have a lower experience in diagnosing pigmented skin lesions.

Cutaneous melanoma is still a skin cancer with the highest mortality despite all the preventive and therapeutic efforts, and this is also despite CM being less common than other non-melanoma skin cancers [8]. Breslow thickness, mitotic activity, and ulceration are associated with an increased risk of metastatic potential and mortality. Therefore, early diagnosis of CM at an initial and curable stage is essential for increasing the survival rate of patients [8]. However, waiting list times for dermatologists have increased and several referred lesions, which are a combination of benign and malignant lesions, can result in a puzzling and inefficient situation where early diagnosis can become challenging for patients who actually do have a malignant lesion. Similar fast-track referral systems, as the one described in our study, have also been implemented in the United Kingdom (UK) in 2000 [9]. When an evaluation of the 2-week rule in the UK was made in 2004, it revealed that the referral system was not working as expected in terms of early recognition and skin cancer diagnosis, because only 12% of the referrals had a potentially metastatic tumor such as squamous cell carcinoma (SCC) or melanoma [10]. As presented in our study, the patients with CM amounted to 312 (56.1%) of all the referred patients. Additionally, a total of 41 (7.4%) of the referred patients in our study were diagnosed with MIS. This means that a total of 353 (63.5%) patients had a malignant or premalignant melanocytic lesion, which demonstrates that it is still desirable to improve the diagnostic accuracy of CM in the primary sector, and this is further highlighted when only considering the patients who were referred without a biopsy, where the diagnostic accuracies for GPs and dermatologists were 29% and 45%, respectively.

By using this referral system, it is understandable and appropriate that some referrals will be made on the basis of doubt, especially if the referring physician is less experienced. Also, some physicians may experience pressure from patients and their concerns of increased waiting times for having routine referrals [11]. In Australia, where there is a high prevalence of skin malignancy, it has been a target to improve awareness and diagnostic skills in Australian GPs for skin malignancies, which has led to improved accuracy and appropriate referral to specialists [12]. In Italy, a formal 4-hour training session on diagnostic and referral accuracy of family doctors in melanoma screening was given to GPs, resulting in improved specificity of suspicious pigmented skin lesion referrals for melanoma from 55% to 73.1% (*P* < 0.001) without significant loss in sensitivity [13]. However, another evaluation in the UK of the 2-week rule after a targeted continuing medical education showed surprisingly that the rate of correctly referred patients with suspected skin malignancies remained low regardless of the education module [14].

Recent evidence has shown that the increase in the incidence of CM is due to a generally higher awareness of skin cancer and the improved strategies for early recognition and diagnosis of suspicious melanocytic lesions. This is resulting in an increasing incidence of early stage melanomas [1]. However, a population-based study presented an increasing incidence of thick CM as well as an increase in incidence of all the other categories of Breslow thickness [15]. Hence, it is essential to ensure that the referrals of patients in FTRS are optimized, to limit unnecessary referrals, in order to gain capacity for the relevant category of patients with CM in the secondary care. Furthermore, distribution of CM over body surface is not only on sun-exposed areas, which indicates the importance of total body skin examination [16]. However, this is rarely performed by the referring physicians, including dermatologists, and thus, it is performed for the first time during patients' visit to the outpatient clinic. Unawareness of the lesion distribution or the time pressure in the clinics may explain why the referring physicians do not perform total body skin examination [16, 17], and this might possibly lead to delayed diagnosis of CM.

A limitation to our study is that this is a single center study based on the figures of one region of the country. However, Denmark is a relatively homogenous country with an even distribution of CM [5], and therefore, the 820.000 inhabitants in Zealand Region are likely representative of the effect and quality of the FTRS. Also, we are aware that we do not have any comparative data from the period before implementation of FTRS. However, since this was a cross-sectional study our objective was to perform a descriptive analysis of the current referral patterns of suspicious skin lesions to our department. Therefore, we conclude that since a definitive screening program for CM does not exist in many countries, it is essential to detect atypical melanocytic skin lesions early and correctly refer these to secondary care, where effective and timely management of CM can be performed. An increase in the level of diagnostic accuracy from the referring physicians is required in order to optimize the precision of the referrals to FTRS. This might be achieved, for example, by formal training sessions or performance of an excision biopsy, leading to an increased diagnostic accuracy and thereby improving and reserving the FTRS to the relevant patients.

## Figures and Tables

**Table 1:** Results of referrals in the fast-track referral system for skin lesions suspicious of melanoma.

	Females	Males	Total	*P* value
*N* (%)	293 (52.7)	263 (47.3)	556	<0.05
Age (mean ± SE)	57 ± 1.0	60 ± 1.0	58 ± 0.7	<0.05
CM	170	142	312	<0.05
In situ (%)	23 (56.1)	18 (43.9)	41	ns
NMSC	13	16	29	ns
Benign	86	85	171	ns

CM: cutaneous melanoma.

NMSC: non-melanoma skin cancer.

**Table 2:** Origin of the referrals in the fast-track referral system and the proportion of diagnosed CM and diagnostic accuracy.

	GP	Derm.	PS	Other	Total
Referrals	88	441	5	22	556
(i) Without biopsy	55	321	0	17	393
CM diagnosed with biopsy prior to referral	31	106	5	5	113
CM verified with biopsy after referral	16	145	0	4	199
CM in total	47	251	5	9	312

CM: cutaneous melanoma.

Derm.: dermatologist.

PS: plastic surgeon.

**Table 3:** Age, gender, and Breslow thickness (%).

Age	Females			Males		
	≤1 mm	>1–4 mm	>4 mm	≤1 mm	>1–4 mm	>4 mm
20–40	(11.8) 20	(1.8) 3	0	(4.2) 6	(2.8) 4	0
41–55	(26.5) 45	(7.6) 14	0	(11.3) 16	(5.6) 8	(0.7) 1
56–70	(19.4) 33	(5.3) 9	(1.8) 3	(24.6) 35	(12.7) 18	(1.4) 2
>70	(11.2) 19	(8.2) 14	(5.3) 9	(16.9) 24	(13.4) 19	(4.2) 6

Total	(68.8) 117	(22.9) 40	(7.1) 12	(57.0) 81	(34.5) 49	(6.3) 9

**Table 4:** Distribution on Breslow thickness of referrals originating from GPs or dermatologists when biopsy is performed prior to and after referral.

Referrals	Breslow thickness			
	≤1 mm	>1–4 mm	>4 mm	Unclassified
GP				
+ biopsy^**∗**^	21	7	2	1
÷ biopsy^**∗**^	9	4	3	0
Dermatologist				
+ biopsy	58	40	4	4
÷ biopsy	89	45	11	0

^*∗*^+ biopsy: biopsy performed prior to referral.

÷ biopsy: biopsy performed after referral.
